# Relationship between psychosocial stress dimensions and salivary cortisol in military police officers**^1^**

**DOI:** 10.1590/1518-8345.1199.2873

**Published:** 2017-04-20

**Authors:** Juliana Petri Tavares, Liana Lautert, Tânia Solange Bosi de Souza Magnago, Angélica Rosat Consiglio, Daiane Dal Pai

**Affiliations:** 2 PhD, Adjunct Professor, Escola de Enfermagem, Universidade Federal do Rio Grande do Sul, Porto Alegre, RS, Brazil; 3 PhD, Full Professor, Escola de Enfermagem, Universidade Federal do Rio Grande do Sul, Porto Alegre, RS, Brazil; 4 PhD, Associate Professor, Departamento de Enfermagem, Universidade Federal de Santa Maria, Santa Maria, RS, Brazil; 5 PhD, Associate Professor, Instituto de Biociências, Universidade Federal do Rio Grande do Sul, Porto Alegre, RS, Brazil

**Keywords:** Worker`s Health, Psychologic Stress, Police, Physiologic Stress

## Abstract

**Objective:**

to analyze the relationship between psychosocial stress dimensions and salivary cortisol in military police officers.

**Method:**

cross-sectional and analytical study with 134 military police officers. The Effort-Reward Imbalance (ERI) Model scale has been used to assess psychosocial stress. Salivary cortisol was collected in three samples. The following tests were used: Student's t-test, Mann-Whitney, ANOVA, Bonferroni, Kruskal-Wallis and Dunn. Pearson and Spearman correlation methods were used, as well as multiple linear regression. Cortisol at night showed an ascending statistical association with the psychosocial reward (p=0.004) and a descending association with the effort-impairment scores (p=0.017). Being part of the Special Tactical Operations Group (GATE) and the diastolic blood pressure explained 13.5% of the variation in cortisol levels on waking up. The sectors GATE, Special Patrol of the Elite Squad of the Military Police and Motorcyclists explained 21.9% of the variation in cortisol levels 30-minute after awakening. The variables GATE sector and Effort Dimension explained 27.7% of the variation in cortisol levels at night.

**Conclusion:**

it was evidenced that salivary cortisol variation was influenced by individual, labor and psychosocial variables.

## Introduction

Police officers, all over the world, are one of the categories of workers at greatest risk of death and exposure to stress^1^ ^-^ ^3^. The exposure and level of stress of the police officers have been pointed out as higher than those of other professionals due to the nature of the activities performed, the low remuneration and the work overload resulting from internal relations in the corporation^1^. Therefore, work can be a source of stress when the worker realizes the imbalance between the high effort made at work and the low reward^4^. 

Being repeatedly subjected to work stress can cause damage to physical and/or mental health when there is an organic vulnerability and an inappropriated form of evaluation and coping with the stressful event. Health problems begin in a discreet and silent manner, and are usually diagnosed late, through physical and mental symptoms.

One of the theoretical models that assesses psychosocial work stress is the Effort-Reward Imbalance (ERI), which is based on the reciprocity between these two constructs in professional life^4^ ^-^ ^6^. Therefore, a person with a greater need for control responds differently to work situations that require a lot of effort and low reward, which causes stress and activates several physiological axes of response to stress in the body^4^ ^,^ ^6^.

The modulation of physiological responses to stress is performed, in addition to the Autonomic Nervous System, by the Hypothalamic-Pituitary-Adrenal axis, which plays a fundamental role in the response to the external and internal stimuli of the stress agents, by regulating the circulating level of glucocorticoids^7^. Cortisol is one of these glucocorticoids secreted in response to stress and found in body fluids. It follows a circadian rhythm both in plasma and urine and saliva: its maximum level occurs in the morning, declines throughout the day, is in low concentration near midnight and increases in the first hours after sleeping^7^.

The cortisol peak on waking up represents a 50-75% increase of its levels within 30 minutes after awakening and has been referred to as a response to cortisol on waking up^8^. After decreasing throughout the day, it returns to low levels in the nocturnal period^9^. The cortisol response on waking up, in addition to serving to mobilize the energy reserves, has been recognized as a way of assessing the expectation in relation to the day, and is quantifiable through the difference between the cortisol levels within 30 minutes upon waking up and the cortisol levels shortly after waking up^8^. Elevated cortisol levels before sleeping may represent an allostatic loading condition^10^. The dosage of free salivary cortisol for the diagnosis of stress has become routine, due to the easy of collection and the low cost, when compared to other methods^7^.

Regarding stress, some studies that have assessed stress in the Brazilian^11^ and Swiss military police officers^12^ and in the elite squad of the Italian military police^2^ ^)^ are highlighted. As for salivary cortisol levels, these were investigated in German^3^, American^13^ and Dutch^14^ ^-^ ^16^ police officers. However, these studies assessed separately the self-perception of stress or the salivary cortisol curve, which allow identifying a gap in the knowledge about the health of the police officers. In this context, it was established as study problem: Is there a relationship between self-perception of psychosocial stress and salivary cortisol in military police officers? 

Interest in this issue is based on the vulnerability of the Brazilian police officers due to the high daily demands in the fight against urban violence, among other sources of stress experienced by these workers seeking to promote the safety of the population. Therefore, this study is justified by the social implications of the collective under study - the military police officers - on the public safety and well-being, as well as on their high risk of illness, which requires attention from health professionals, especially from nursing professionals since they play a key role in health education and promotion practices.

In view of the above, the objective of this study was to analyze the relationship between psychosocial stress dimensions and salivary cortisol levels in military police officers. The military police is a permanent and regular part of the public security system of the State of Rio Grande do Sul and responsible for ostensive policing, preservation of public order, external custody of prisons and military judicial police. Therefore, it is up to the military police officers to ensure compliance with the law, maintenance of public order, technical management of high-risk situations, environmental protection and external custody of state prisons, among others. 

## Material and methods

**Figure 1:**
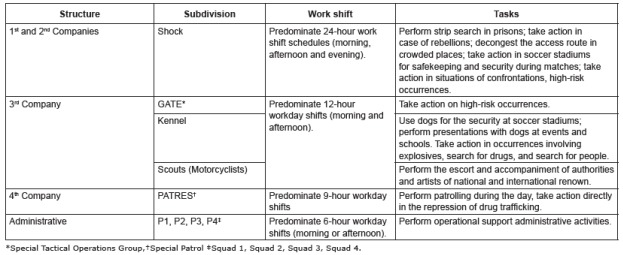
Description of the structure of the 1^st^ Special Operations Battalion of the state of Rio Grande do Sul with its respective subdivisions, work shifts and tasks performed by the military police officers. Porto Alegre, RS, Brazil, 2013

This study consisted in an observational and analytical investigation carried out in the Special Operations Battalion (BOE) of the Military Police in a municipality in the state of Rio Grande do Sul. BOE is divided into four companies (CIAs) which, in turn, have subdivisions. Figure 1 shows the CIAs, subdivisions within each CIA, work shift and tasks.

The study population consisted of 416 BOE military police officers, of which 317 were in active service. The subjects in active service were included in the sample, with age range from 18 to 65 years. The exclusion criteria were: police officers who had worked less than one year in the corporation (54 police officers); female police officers, since they represent less than 5% of the BOE population and develop a different cortisol response in comparison with male sex; and those on corticosteroids^7^. Based on these criteria, the eligible population of this study was 263 military police officers. Of these, 258 answered the data collection instrument and 134 collected the three samples of saliva. 

The sample size calculation was performed using G*Power *software* Version 3.1.2 (2009). It was considered the association between the factor under study and 0.3 as outcome, multiple linear correlation model with eight predictor variables and an effect size f^2^=0.15, with statistical power levels from 90 to 94%, and a significance level of 5%. The calculated sample size consisted of 134 BOE military police officers. 

The data were collected from June 2012 to July 2013, at the headquarters of the Special Operations Battalion. Pre-trained PhD, Master's and Nursing undergraduate students performed data collection. The anthropometric measurements were carried out^17^ by the researchers at the time the questionnaire was applied.

For saliva collection, oral and written instructions were provided in order to: avoid food intake, drinking, smoking, and for not brushing teeth 30 minutes before each collection. These instructions were given in more detail as follows: 1. Saliva collection should be carried ou at three times: on waking up, 30 minutes after waking up and before bed; 2. For a period of 30 minutes prior to collection, avoid food intake, drinking (other than water), or smoking; 3. Fasting is not necessary, however, after dinner wait at least 3 hours to collect the third sample of saliva; 4. Do not exercise for 1 hour prior to collection; 5. Immediately before collection it is advisable to wash the mouth with water; 6. Collection is not recommended in cases of oral lesions with active or potential bleeding; 7. Not having undergone dental treatment in the last 24 hours; 8. Not having brushed the teeth in the last 3 hours in order to avoid gingival bleeding; 9. Keep the tubes of the three saliva samples in the refrigerator if possible; 10. Deposit the three samples of collected saliva in the next working day, in the same place where the questionnaire was filled out.

Information on sociodemographic data was collected, such as: age, marital status; work information: work sector, post/rank, length of service, work elsewhere, overtime, weekly workload, daily work shift, work shift, work pace; lifestyle information: smoking, sleeping hours, health problems, psychoactive beverages (alcohol, mate, coffee, Coca-Cola); anthropometric and cardiovascular measurements (blood pressure - BP, weight, height, body mass index, waist circumference, hip circumference).

Psychosocial stress was assessed using the Effort-Reward Model (ERI) scale, which contains 16 questions of the long version^6^. Dr. Johannes Siegrist's research group shortened the scale in order to facilitate its use. In this study, the recommendations of its creators were followed, with the removal of 16 questions from the long version validated in Brazil^5^. The short version validated in Brazil^18^ ^)^ was considered reliable (α=0.703). For the effort dimension, questions 1, 2 and 3 were used; for the reward dimension, questions 7, 11, 12, 13, 15, 16 and 17 (with 11, 12 and 13 being reverse questions); and for the dimension of over-commitment, questions 18 to 23. This said, in this study, it was carried out the factorial analysis of the scale, in which the questions were grouped into two factors: reward and effort- impairment. For purposes of analysis, the ratio between effort-impairment and reward dimensions was calculated by means of the factorial scores of the scale, and the data of the two dimensions were analyzed as continuous. 

Salivary cortisol was collected at three times: on waking up, 30 minutes after waking up and before bed (night), with at least one milliliter of saliva in each sample, collected in Salivettes(r) tubes with cotton swabs. These three moments for the collection of cortisol have been used in many epidemiological studies^19^, taking into account factors such as cost and benefit, as well as adherence to the research and full return of the collected material. 

For salivary cortisol measurement, the saliva samples were centrifuged (1500 rpm/3 min), frozen and maintained at -20°C, to allow precipitation of proteins and mucins, and submitted to radioimmunoassay analysis with the *^_Cortisol Coat-A-Count(r) Kit_^* (*Siemens Medical Solutions Diagnostics, Los Angeles*, California, USA). The sensitivity was 0.09 nmol/L; with the use of the Gamma C 12(r) Counter (EURO-DPC)^20^. The reference values determined by the Laboratory (LabVitrus) where the test was carried out were: for morning cortisol (8-9h), from 13.5 to 23.5 nmol/L; and for nocturnal cortisol (22-23h) from 1 to 2.9 nmol/L.

Data were analyzed using the *Statistical Package for the Social Sciences* (SPSS(r)) version 18.0 for Windows. The Shapiro-Wilk test for normality was used to verify the distribution of variables, asymmetry and kurtosis values. The cortisol variable (outcome) was submitted to a mathematical transformation (square root) and the *outliers* were removed for the multivariate statistical analysis of data^21^. Student's t test was used to determine the association between variables with symmetrical distribution, and the Mann-Whitney test for asymmetric distribution. The parametric variables, with three groups or more, were submitted to an ANOVA variance analysis, the *post hoc* differences to the Bonferroni test and, the non-parametric variables to the Kruskal-Wallis and Dunn.

In order to evaluate the relationship between the factorial scores obtained in the ERI scale and the cortisol levels, the bivariate Pearson correlation (symmetric variables) and the Spearman correlation (asymmetric variables) were performed. Multiple Linear Regression (*Stepwise*) was used to analyse the association among the variables. In the regression model, the variables that showed association with cortisol were selected, with a 75% confidence level (p ≤ 0.25). Data with a two-sided "p" values less than 0.05, or with a 95% confidence interval were considered as statistically significant differences.

The Research Ethics Committee (CEP) of the Federal University of Rio Grande do Sul approved the research under protocol number 19785. The ethical principles were respected in accordance with the precepts established by Resolution 466/12 of the National Health Council for research on human beings^22^. All participants signed the Informed Consent Form.

## Results

Regarding the sociodemographic characteristics, lifestyle and anthropometric and cardiovascular measurements, the mean age was 35 (29-34) years, the majority of the police officers were married or had a partner (75.2%), non-smokers (92.5 (73.1%), with systolic (117-130 mmHg) and diastolic (70-84.7 mmHg) blood pressure within the normal parameters, on psychostimulants (84.3%), the average body mass index (BMI) ranged from 24.9 to 29.1 kg/m² and they presented from 6 to 7 hours of sleeping per night.

As for the work sector of the military police officers, most belonged to Shock (41.8%, n=56), followed by GATE and administrative sectors, both with the same percentages (20%, n=14.9), PATRES (16%, n=11.9), Kennel (9%, n=12) and Motorcyclists (7.5%, n=10). 

The average length of service of the military police officers in the institution was 9 (4; 23) years, and 7 (3-20) years in the sector. The average weekly workload was 43 (40-50) hours, and 12 (8-12) hours per day.

The highest percentage of military police officers corresponded to the rank of soldier (70.7%, n=94) and worked overtime (72%, n=95). Additionally, 56 (41.8%) worked during the day and night periods, with no 24-hour shift schedule and with 48 hours rest; 51 (38.3%) worked elsewhere and 67 (50.4%) considered the number of people in the work scale as insufficient.

The average values of cortisol levels of the BOE military police officers were 11.7 (8.8-15.1) nmol/L on waking up, 12.5 (9.5-16.9) nmol/L 30 minutes after awakening and 3.9 (3.2-4.8) nmol/L at night. The variables age (r=-0.174), diastolic BP (r=-0.178), weight (r=-0.177), waist circumference (r=-0.226) and hip circumference (r=-0.200) were negatively associated to cortisol levels on waking up (p<0.05). 

Cortisol levels at night were negatively correlated with the variable waist circumference (r=-0.216, p=0.012) and positively correlated with the cortisol levels of non-smoker police officers and those with no health problems compared to those who had problems and were smokers (p<0.05). In assessing the association between work sector and salivary cortisol, the Special Tactical Operations Group (GATE) showed the highest averages of cortisol levels on waking up compared to motorcyclists and shock (p<0.01). GATE also showed the highest averages of cortisol 30 minutes after awakening compared to motorcyclists, PATRES, shock and administrative sectors (p<0.01), and the highest average values of cortisol at night in relation to all other sectors (p<0.01). 

The cortisol levels on waking up were negatively associated with the weekly workload (r=-0.176, p=0.042). Cortisol levels at night were significantly associated with the variable number of people in the work scale, so that police officers who considered the number of people as sufficient presented the highest average values of cortisol levels at night compared to those who evaluated the number of people as insufficient (p=0.023). The other sociodemographic, lifestyle, anthropometric and cardiovascular variables were not significantly associated with cortisol levels (p>0.05). 

**Figure 2:**
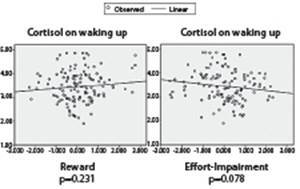
Linear regression for cortisol levels on Waking up and Psychosocial Stress Dimensions. Porto Alegre, RS, Brazil, 2013

 The regression lines for cortisol levels on waking up and cortisol levels 30 minutes after awakening presented a downward trend in relation to the effort-impairment scores, but no significant difference (p>0.05) was observed in the regression analyzes (Figures 2 and ^3^).

**Figure 3:**
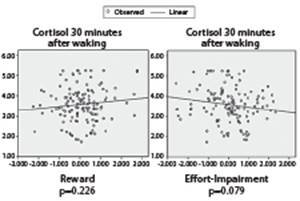
Linear regression for cortisol levels 30 minutes after awakening and Psychosocial Stress Dimensions. Porto Alegre, RS, Brazil, 2013

**Figure 4:**
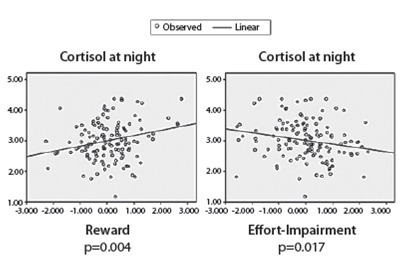
Linear regression for cortisol levels at night and Psychosocial Stress Dimensions. Porto Alegre, RS, Brazil, 2013

In the bivariate analysis among the reward, effort-impairment and cortisol levels at night, it was observed a positive correlation between cortisol at night and reward (r=0.249, p<0.001) and a negative correlation between cortisol at night and effort-impairment (r=-0.206; p=0.005). The regression line for cortisol at night and reward showed an ascending statistical association (p=0.004) and a descending statistical association for cortisol levels at night and effort-impairment scores (p=0.017) (Figure 4).

Table 1 shows the results of the multivariate analysis for the variables included in the final model. 

**Table 1:** Multivariate linear regression model, output variables of the final model related to salivary cortisol on waking up, 30 minutes after awakening and at night. Porto Alegre, RS, Brazil, 2013

	Beta standardized	β (95%CI)	p value	R**^2^** *
Cortisol on waking up				
GATE**^†^**	0.322	0.578 (0.283; 0.874)	<0.001	0.135
Diastolic BP**^‡^**	-0.190	-0.013 (-0.025; -0.002)	0.023	
Cortisol 30 min				
GATE**^†^**	0.354	0.861 (0.478; 1.243)	<0.001	
PATRES**^§^**	-0.187	-0.499 (-0.919; -0.08)	0.020	0.219
Motorcyclists	-0.175	-0.578 (-1.093; -0.063)	0.028	
Cortisol at night				
GATE**^†^**	0.493	0.428 (0.298; -0.558)	<0.001	0.277
Effort-Impairment	-0.183	-0.055 (-0.100; -0.011)	0.016	

*Coefficient of determination. Stepwise selection method was used.

†Special Tactical Operations Group

‡Blood pressure

§Special Patrol

It was observed that the variables GATE and diastolic BP had the greatest direct and inverse influence, respectively, on cortisol levels on waking up. Belonging to the GATE sector increases the outcome by 0.32 units, and having high diastolic BP causes a decrease of 0.19. These variables explain 13.5% of the variation of cortisol levels on waking up.

Regarding cortisol 30 minutes after awakening, the variables with the greatest influence are GATE, PATRES and Motorcyclists. The first one has a direct relation, and the others ones have an inverse relation. The GATE sector increased by 0.35 units such outcome and the PATRES and Motorcyclists sectors caused a decrease of 0.18 and 0.17 units, respectively. These three variables explain 21.9% of the variation in cortisol levels 30 minutes after awakening.

Cortisol levels at night is directly influenced by the variable GATE sector, which causes an increase by 0.49 units, and is inversely influenced by the variable effort-impairment, which causes a decrease of 0.18 units in the cortisol outcome at night. These variables explain the 27.7% variation of cortisol levels at night.

## Discussion

Data analysis showed that the individual variables of BOE military police officers, such as age, diastolic BP, weight, waist circumference and hip circumference were negatively related to cortisol levels on waking up. These findings are similar to what occurred in the study with 373 Police officers from Buffalo, USA, in which the reduction of cortisol response to a challenge was an indicative of Metabolic Syndrome, after regression adjustment for age and sex^23^. Age, which was also negatively related to cortisol levels on waking up in the current study, was positively correlated with these levels (r=0.14, p<0.001) in an investigation with police officers in four US cities, and negatively correlated with Peritraumatic Stress (r=-0.13, p<0.05) in workers with 12 months of service in this function^24^. These findings suggest that the low production of cortisol on waking up may be due to Metabolic Syndrome and/or stress, an element commonly found in the work of this professional category.

In this study, cortisol levels at night, in turn, in addition to being negatively related to waist circumference, showed the highest scores for non-smokers police officers and for those without reports of health problems. The low variation of cortisol levels during the day, that is, low cortisol levels in the morning and high at night, may be associated with flattening of the cortisol curve, which indicates signs of chronic stress^25^. These findings lead to inferring that police officers with the lowest abdominal circumferences, who did not smoke and without health problems were possibly more active and committed to work, finding it difficult to "disconnect" from work at night. On the other hand, stress and hyperactivation of HPA axis can influence the increase of adipose tissue and vice versa, so that obesity can contribute to the consolidation of a state of chronic stress^23^.

Police officers of the GATE sector presented the highest salivary cortisol scores in the three measurements. Professionals of this sector perform functions similar to those of the Special Police Operations Battalion of Rio de Janeiro (BOPE)^26^. They correspond to the operational elite of the Military Police of Rio Grande do Sul and perform special operations in situations where the risk to life is greater than in other sectors, such as: kidnapping, bomb disarmament, assaults, among others. In general, these occurrences cause psychological tension, difficulties, discomforts arising from atmospheric conditions and deprivation of the basic needs such as hunger, thirst, sleep, fatigue, among others. Therefore, the work performed by these workers requires improved and constant training as well as physical and mental ability to perform their work. As such, increased cortisol levels on waking up may be related to their affinity for work, and increased cortisol levels at night may be to assure readiness, since even if they do not work daily on the night shift, they may be requested at any time, remaining in state of readiness. High cortisol levels on waking up were also identified among firefighters assessed after exposure to a highly stressful and potentially traumatic event, and associated with physiological reactivity for tasks involving high demand and fear conditioning^27^.

Thus, although all sectors of BOE have the mission of maintaining public safety and face the same working conditions, the work at GATE, Kennel and Motorcyclists sectors is quite different and is a reference for the entire state, which may allow a closer identity thereof.

The weekly workload was negatively related to cortisol on waking up, ie, the higher the workload, the greater the overload, the more frequent the exposure to stressors at work, the less cortisol response on awakening. However, police officers who considered the number of people on the job scale as sufficient had the highest average of cortisol levels at night when compared to those who rated it as insufficient. This data may be intrinsic to the mobilization of these workers towards work, surpassing their physical and mental capacity, without noticing that the overload may be due to the lack of employees and the excessive overtime worked. For some professionals working in/with dangerous activities, such as the police officers, a certain amount of stress is positive and necessary so that the work is achieved^1^. This is a characteristic of these professionals, who face, in addition to the direct demands of the work, hierarchy and discipline, other demands related to the organization of the work in view of the expectations of the society^11^. 

However, many suffer from such work regime. In the opinion of the military police officers who patrol the streets, having two jobs, working night and day, spending 12 hours on the street, on alert, working under pressure and sleeping little, affect their quality of life and health in a counterproductive way^26^.

In addition to the association between cortisol levels and individual and work variables, some studies have experimentally identified alterations in salivary cortisol levels in stress situations^3^ ^,^ ^13^ and reported that well-conducted simulations can help to improve worker performance and reduce stress. In a simulation of school shootout conducted by the German police, cortisol levels were higher at the beginning of training and decreased subsequently^3^. In the assessment of the decision-making capacity of US police officers, in a simulated work-stress situation, the higher cortisol variation was associated with a lower number of errors (better performance) to armed targets and with a better perception of stimuli related to threat and surveillance^13^. 

The association between cortisol at night and the reward dimension assessed through ERI scale may represent the expectation of these police officers in relation to their work and the state of alert in which they remain in order to achieve it. This ERI subscale is about the respect and prestige from superiors and colleagues, the guarantee of employment, prospects of of career advancement, among other elements present in the military corporations. On the other hand, the inverse association between cortisol at night and effort-impairment dimension may be due to the structure and hierarchy of the police officers job. Time pressure, work overload and excessive responsibilities are components of most questions of this scale dimension, and are part of the daily work of these professionals and can therefore cause chronic stress and consequent inhibition of cortisol production. 

A research with a population of 2,126 workers in London on the relationship between stress at work and daytime salivary cortisol identified that the low reward and high scores of ERI were associated with a shallower slope in the cortisol curve throughout the day, represented by decreased cortisol levels in the morning and high levels at night^28^. Such results are similar to those found in this study. However, differently, the variable reward of BOE workers was related to high cortisol levels at night. Perhaps the work at BOE demands a greater mobilization of the police officers to achieve the desired reward compared to the work of the Londoners evaluated. The reward assessed by the ERI Model also evaluates the possibility of future career advancement and being benefited from these career advancements implies greater responsibility, demands from the Government and society and goals. This, as a result, keeps the police officer's HPA overstimulated.

It was evidenced that GATE sector was the variable with greater influence in the three levels of cortisol. In addition, it is relevant to mention that effort-impairment was negatively associated with cortisol at night, which may indicate that effort is something positive for this sample, perhaps due to their affinity for the job.

A study with police officers and firefighters from Amsterdam on postraumatic stress, hypervigilance and salivary cortisol levels found that sex, smoking and negative life events accounted for 10% of cortisol variation (Adj. R2=0.102, se = 0.52 , F = 53.97, p <0.001, df = 4)^14^. Both in the present study and in the Dutch one, salivary cortisol variation was influenced by individual and labor variables, but the salivary cortisol levels of BOE police officers was also altered by the effort-impairment psychosocial variable.

The data of the study at issue show a possible flattening of the salivary cortisol production curve of BOE military police officers and may indicate a possible exposure to chronic stress, which, together with weight, blood pressure and other characteristics of this sample constitute a risk of falling ill for these professionals. Therefore, nursing interventions are essential in order to empower individuals to take responsibility for their individual and collective health in order to maintain it and consequently better face the stress inherent to their profession. To this end, nursing has different care technologies that aim to develop skills for self-management of health care in order to promote a healthy state in individuals. Interventions of this nature have a positive impact on the number and length of Sick Leave and they minimize the risks to the health, indirectly contributing to public safety by promoting the health of military police officers. 

One of the limitations of this study is the selection of the sample by convenience, since all BOE police officers in active service were invited to participate in the study, but 134 deposited the collected saliva, a fact that may have caused healthy participant bias. In addition, each sample corresponds to a specific and relatively small unit of the military police, when compared to that of the whole state. Therefore, the results of this research may not be generalized to all military police officers or those with varying degrees of occupational exposure. Another limitation is the cross-sectional design of the study, which exposes only a single view of the assessment of physiological and labor stress and does not allow establishing a cause-effect relationship. In this context, those who were on sick leave or away from work may constitute a relevant portion of the police officers exposed to stress and its complications. 

This study represents a gain in knowledge since it identified an association between psychosocial stress dimensions and a biological stress marker in military police officers. Furthermore, it is highlighted the peculiarity of the Special Tactical Operations Group (GATE) as the sector most exposed to stress, which allows inferring that this job specifications have implications on the health of these military police officers. 

## Conclusion

It was concluded that the effort-impairment dimension of the psychosocial stress model influenced the variation of cortisol levels at night, as well as belonging to GATE sector. The variables diastolic blood pressure and to belong to GATE sector explained the model of the cortisol levels on waking up. In addition, to belong to GATE, PATRES and Motorcyclists sectors explained the variation in cortisol levels occuring 30-minute after awakening.

It is highlighted the health promotion of the military police officers as a challenge for the public health field, especially with regard to the studies and practices on the health of the workers. In this context, nursing finds potential space for actions aiming at the prevention, promotion and health surveillance, considering this group of workers and their relevance for the preservation of safety and public welfare with an evident implication on human health.

Longitudinal studies are suggested in order to verify the cause and effect relationship between exposure and outcome, as well as the measurement of other variables that may interfere with the alteration of cortisol levels in military police officers.
